# 3-D changes in tooth position after treatment with mandibular advancement devices -a retrospective study in a non-university setting

**DOI:** 10.1186/s12903-025-05914-6

**Published:** 2025-04-11

**Authors:** Sabine S. Linsen, Alexander Meyer, Nikolaos Daratsianos, Anton Kares

**Affiliations:** 1 Department of Prosthodontics, Preclinical Education and Dental Material Science, University Hospital Bonn, Welschnonnenstr. 17, 53111 Bonn, Germany; 2 Private Practice, Friedrich-Ebert-Str. 21, 42719 Solingen, Germany; 3 Department of Orthodontics, University Hospital Bonn, Welschnonnenstr. 17, 53111 Bonn, Germany

**Keywords:** Mandibular advancement devices, 3D tooth movement, 3D digital models, 3D superimposition, Dental side effects, Oral appliances, Sleep apnea, MAD design

## Abstract

**Background:**

While mandibular advancement devices (MADs) are effective in reducing obstructive sleep apnoea (OSA)-related events, they can also cause occlusal changes. Three-dimensional (3D) techniques have rarely been used to evaluate the dental side effects. The aim of this study was to evaluate 3D tooth movement in patients with sleep apnea and to assess the effects of two designs: side wing and side thrust.

**Methods:**

Virtual models of dental casts were superimposed at baseline (T0), after 11.9 ± 7.1 months (T1), and 31.9 ± 25.4 months (T2), evaluating 3D tooth movement, overjet, and overbite. Teeth were grouped into anterior (canine to canine) and posterior (bilateral first premolar to second molar) segments. Normality was checked (Shapiro-Wilk). T-tests, ANOVA, and regression analyses assessed differences in tooth movement, overjet/overbite changes, and influencing factors (*P* <.05).

**Results:**

A total of 58 patients diagnosed with obstructive sleep apnea (mean age: 50.7 ± 12.4 years; 44 males) were included. Observation period 1 (T0 and T1; OP1, *n* = 58) involved 28 wing (Somnodent flex and fusion) and 30 thrust (IST + and classic, Narval CC, Hamburger UPS) appliances, while observation period 2 (T0 and T2; OP2, *n* = 21) included 12 and 9, respectively. Over the treatment period, overjet and overbite decreased (*p* ≤.06), with increased maxillary palatal inclination, distal tooth translation in the anterior/posterior segments, and palatal movement of the anterior segment (*p* ≤.037). Wing appliances demonstrated greater reductions in overjet and overbite (*p* ≤.06), maxillary posterior segment extrusion (*p* =.003), and mesio-buccal translation in mandibular segments (*p* ≤.023) during OP2. Regression analysis indicated that wing appliances significantly influenced overjet and overbite in OP2 (*p* ≤.047).

**Conclusions:**

MAD therapy resulted in progressive dental changes, including mesio-occlusion and anterior open bite, and appeared to be tilting of the entire row rather than physical movement, with wing appliances showing greater, although clinically insignificant, effects.

**Trial registration:**

Registry: drks.de; Name: Changes in tooth position after treatment with mandibular advancement devices as part of the treatment of sleep-related breathing disorders; URL: https://drks.de/search/en/trial/DRKS00034679 Identifier: DRKS00034679, date of registration: 17. July 2024.

## Background

Obstructive sleep apnea (OSA) is a common sleep-related breathing disorder caused by upper airway collapse during sleep, with a prevalence of 2–14% [1]. Continuous positive airway pressure (CPAP) is the primary treatment, while mandibular advancement devices (MADs) are a second-line option. MADs offer several advantages: They are minimally invasive, silent, portable, and promote better adherence [2]. 

MADs work by advancing the mandible to increase upper airway volume, reducing airway collapse [3]. Studies show MADs are as effective as CPAP for mild to moderate OSA (AHI (apnea-hypopnea index) 5–30), and a viable option for severe OSA (AHI > 30) in CPAP-intolerant patients. Reported success rates range from 47.7 to 75.0% [4–9]. 

While MADs effectively reduce OSA-related events, they can also cause occlusal changes, mandibular repositioning, and side effects like TMJ discomfort, myogenic issues, hypersalivation, and tooth sensitivity [10]. The most common side effect is undesirable dental movement, due to distally directed forces on the upper teeth and anteriorly directed forces on the lower teeth [11]. Tooth movement, especially in the anterior region, often appears as palatal inclination of maxillary incisors and labial inclination of mandibular incisors, reducing overjet and overbite [12–15]. In the posterior region, distal movement of the maxillary teeth [10, 12], mesial displacement of the mandibular segments [14, 16–18], and posterior open bites [14, 16, 19–22] have been reported. These changes are influenced by appliance design, degree of mandibular advancement, and duration of use [5, 19, 23–25]. 

Previous studies have examined long-term MAD-induced dental changes using plaster casts [26, 27] or cephalometric radiographs [17, 18, 20, 28]. Although 3D surface scanners are now standard in orthodontics and offer precise assessments of tooth movement [29], they have rarely been used to evaluate MAD-related effects.

This study aims to assess 3D maxillary and mandibular tooth movements, including tipping and displacement, and to examine the relationship between appliance design and adverse dental changes in patients treated with MADs for sleep apnea. The null hypothesis stated that the dental arch would undergo a tilting movement rather than a translation or bodily movement, and this is primarly dependent on the splint’s propulsion.

## Methods

This retrospective study analyzed model casts of patients treated with MAD by a dental sleep medicine dentist (A.M.) in a non-university setting between December 2006 and October 2020.

Inclusion criteria were age > 18 years, pre-treatment and post-titration polysomnography (PSG)/ polygraphy (PG), complete pre-treatment and 1-year follow-up stone cast sets (orthodontic socketed models) with full reproduction of maxillary palatal folds. Exclusion criteria included central sleep apnea or other coexisting sleep disorders, lack of adherence (MAD < 50% of nights), and digital impression models. The study was approved by the University of Bonn Ethics Committee (309/20).

### Data collection

**Fig. 1:**
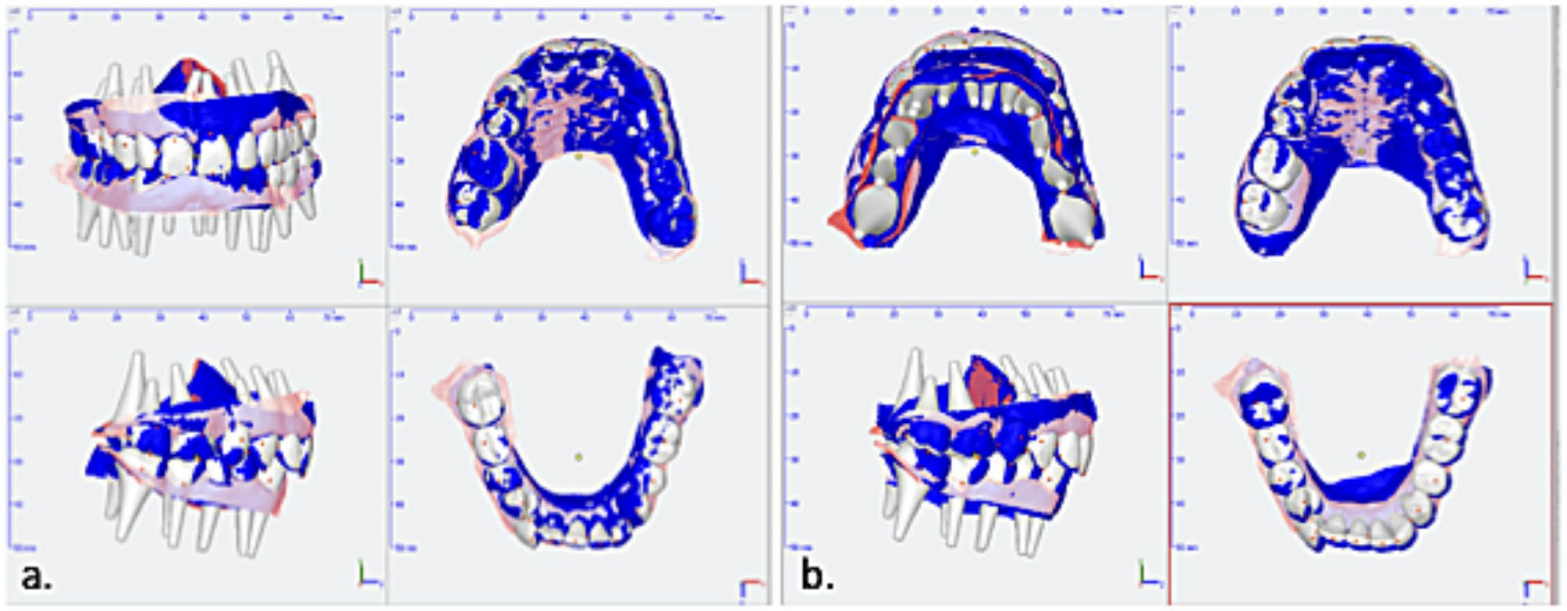
Analysis of tooth movement in the 3D software. Caption: Comparison of tooth position from baseline (white) and after observation periods 1 (**a**) and 2 (**b**) (blue) in a representative case

Patients’ demographic medical records, sleep parameters, and stone casts were collected at baseline (T0), 1 year (T1), and after > 2.5 years (T2). Stone casts were digitized and superimposed for 3D tooth movement analysis. The baseline model was digitally superimposed on the T1 model (observation period 1 (OP1), T0-T1) and on the T2 model (observation period 2 (OP2), T0-T2) to measure tooth position and migration changes (Fig. 1).

### Primary outcome measures

3-dimensional (3D) maxillary and mandibular tooth movements after 11.9 ± 7.1 months (T1) and 31.9 ± 25.4 months (T2) MAD therapy.

### Secondary outcome measures

Relationship between MAD design (wing or thrust) and adverse dental changes.

### Treatment protocol

**Fig. 2:**
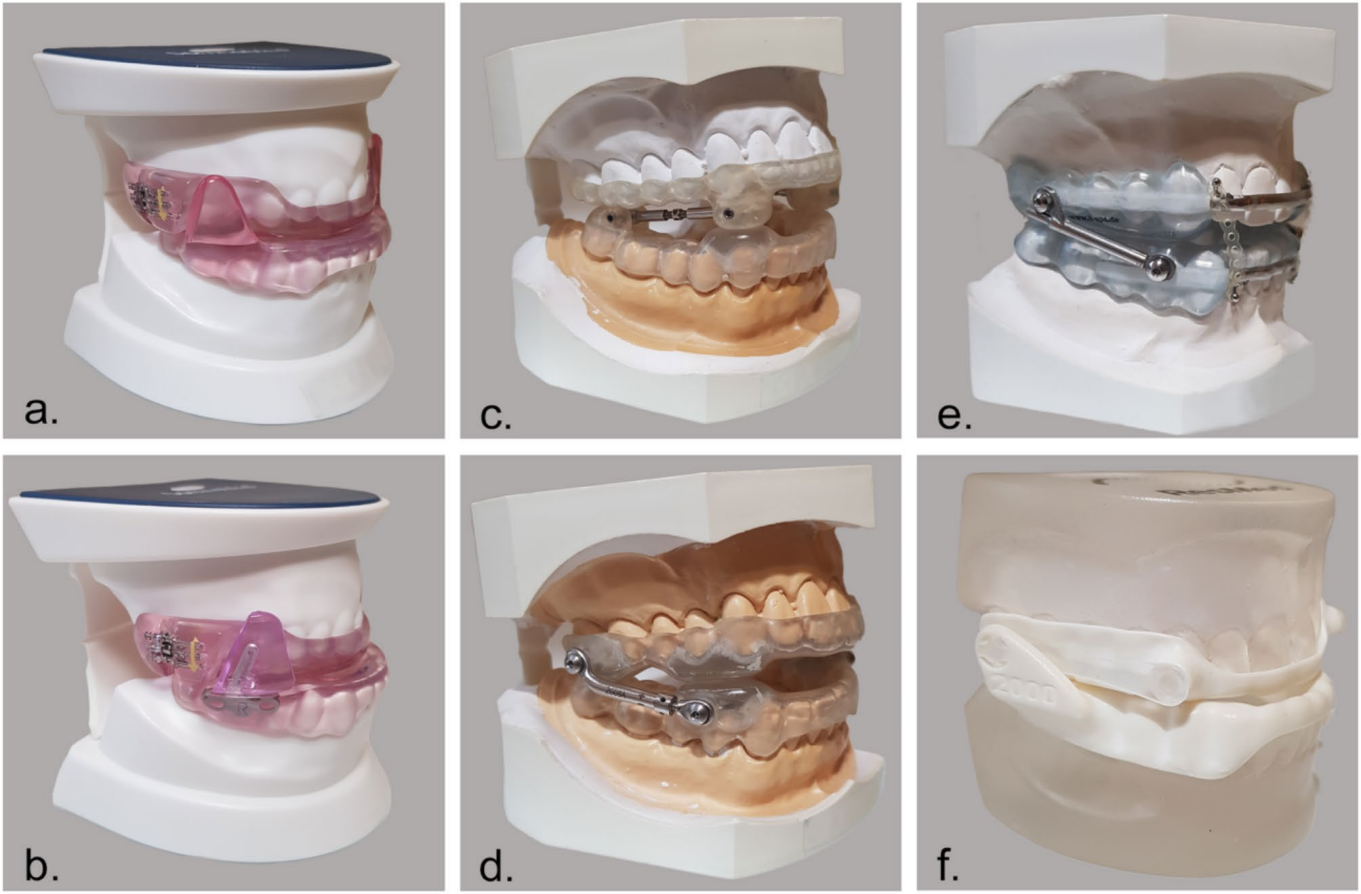
MAD designs. Caption: Lateral wing (Somnodent flex (**a**) and fusion (**b**) (SomnoMed AG, Zug, Switzerland) and lateral thrust (IST+ (**c**) and classic (**d**) (Scheu-Dental, Iserlohn, Germany), Hamburger UPS (**e**) SleepLikeMe-Medical GmbH, Henstedt-Ulzburg, Germany)) Narval CC (**f**) ResMed Healthcare Germany, Martinsried, Germany)

After OSA diagnosis, patients initially received a test MAD made from soft elastomeric material (modified Lyon appliance, Fig. 2) at about two-thirds maximum mandibular protrusion for up to 6 weeks. Following a 2-week familiarization, device efficacy was evaluated by PSG or PG. Patients who responded positively were fitted with a definitive MAD.

All definitive MADs were bimaxillary, two-splint devices adjustable by at least 5 mm anteriorly and 1 mm posteriorly. Two designs were included: thrust mechanisms (IST + and classic (Scheu-Dental, Iserlohn, Germany); Narval CC (ResMed Healthcare Germany, Martinsried, Germany); Hamburger UPS (SleepLikeMe-Medical GmbH, Henstedt-Ulzburg, Germany)) and wing mechanisms (Somnodent flex and fusion (SomnoMed AG, Zug, Switzerland)), as shown in Fig. 2. Final MAD efficacy was confirmed by PSG or PG, after titration adjustments at follow-up visits (1, 4, and 12 weeks) to optimize mandibular protrusion and symptom improvement.

### Model analysis

Stone casts (class IV super hard stone; Picodent, Wipperfürth, Germany) were made from alginate impressions (Kaniedenta, Herford, Germany) of the maxillary and mandibular arches at T0, T1 and T2. The casts were trimmed, aligned using bite registrations in habitual occlusion (Kanibite Extreme, Kaniedenta, Herford, Germany), placed in orthodontic base formers and socketed. Casts were scanned using an optical structured light scanner (model scanner S600 Arti, Zirkonzahn GmbH, Gai/Südtirol, Italy) with < 10 μm accuracy, generating STL (standard tessellation language) files for 3D analysis using OnyxCeph (Image Instruments GmbH, Chemnitz, Germany).

#### 3D processing software

STL files from T0, T1, and T2 were imported into OnyxCeph for segmentation, superimposition, and tooth movement measurement.

##### Segmentation

After aligning all digital models in OnyxCeph, each tooth in the T0 model was manually identified and assigned using the ‘Synthetic Roots Short’ tool.

##### Superimposition

Using the ‘Inspect 3D’ tool, models (T0 vs. T1, T0 vs. T2) were superimposed. The best fit method was used with reliable reference regions. For maxillary models, palatal folds, circumferential gingiva (0.5–1 cm from the teeth), and the area along the palatal suture (1–2 cm) were used as reference landmarks [30–32]. After aligning all models in the same coordinate system, tooth position changes between T0 and T1 (observation period 1, OP1), and T0 and T2 (observation period 2, OP2) were assessed. For mandibular models, alignment was done using occlusal contacts and maxillary palatal folds due to the lack of stable anatomical reference points [33]. 

##### 3D tooth movement

Tooth movements between T0 and T1 (OP1) and T0 and T2 (OP2) were evaluated for angulation, inclination, rotation, and displacement along defined axes. OnyxCeph uses a non-Cartesian (oblique-angled) coordinate system:

###### Angulation (°)

Rotation around the vestibular axis. Increasing angulation moves the incisal landmark mesially and the apex landmark in the opposite direction.

###### Inclination (°)

Rotation around the ‘m/d-axis’ (mesial versus distal arch points). Increasing inclination moves the incisal landmark buccally and the apex in the opposite direction.

###### Rotation (°)

Rotation around the ‘tooth axis’ (mid point versus apex). Increasing rotation moves the labial landmark mesially.

###### Displacement (mm)

Displacements along the crown coordinate axes.

- Root axis displacement is positive in the apical direction (intrusion).
- M/d axis displacement is positive in the mesial direction.
- Vestibular axis displacement is positive in the vestibular direction.

Tooth movements were grouped into anterior (canine to canine) and posterior (bilateral first premolar to second molar) segments.

#### Overjet and overbite

Overjet and overbite were calculated as mean values based on the upper central incisors at T0, T1, and T2. Overjet was defined as the horizontal distance from the mesial end of the maxillary central incisor’s incisal edge to the mandibular central incisor’s labial plane. Overbite was defined as the vertical overlap between the two.

### Statistical analysis

Data were analyzed using SPSS (version 29 for Windows, SPSS Inc., Chicago, IL, USA) following Strobe guidelines [34]. Data are presented as mean and standard deviation (SD).

The Shapiro-Wilk test checked normality of each data set. Differences in tooth movement between OP1 and OP2 were assessed with t-tests or Wilcoxon tests, while overjet/overbite differences across time points (T0, T1, T2) were analyzed using one-way ANOVA with post hoc Bonferroni correction. Mann-Whitney U tests compared wing and thrust designs. Multiple linear regression without weighting assessed the impact of variables like missing teeth, initial PSI, initial overjet/overbite, MAD design, and protrusion on overjet and overbite changes. Statistical significance was set at *P* <.05.

## Results

Plaster casts from 58 patients (44 males, 14 females) aged 50.7 ± 12.4 years were analyzed. Demographic data, overjet and overbite measurements, and impressions were collected at T0 (baseline; *n* = 58), T1 (approximately 1 year; 11.9 ± 7.1 months; *n* = 58), and T2 (approximately 2.5 years; 31.9 ± 25.4 months; *n* = 21) of MAD therapy (Table 1). PG/PSG exams were conducted close to each time point.

**Table 1:** Demographic / clinical information, PSG / PG data, mandibular advancement device specifications

variables	T0		T1		T2		ANOVA/ Friedman*
	*n*	Mean (SD)/%	*n*	Mean (SD)/%	*n*	Mean (SD)/%	
**demographics / clinic variables**							
male/female ratio	58	44/14	58	44/14	21	16/5	
age (years)	58	50.72 (12.40)	58	51.81 (12.32)	21	54.1 (12.74)	
treatment time (months)			58	11.94 ( 7.06)	21	31.92 (25.44)	
missing teeth maxilla mandible	58	2.76 ( 1.73) 2.60 ( 1.60)	58	2.76 ( 1.73) 2.6 ( 1.6)	21	2.86 ( 1.56) 3 ( 1.64)	
PSI (worst sextant)	58	2.10 ( 0.87)	46	1.72 ( 1.10)	20	2.10 ( 1.07)	0.345
**PSG / PG data**							
AHI (no./hour)	56	18.47 (13.03)	48	8.94 ( 8.97)	12	5.09 ( 4.02)	**0.001**
minSaO_2_ (%)	54	87.02 ( 4.81)	46	88.28 ( 6.86)	12	89.83 ( 4.09)	**0.012**
**advancement device specification**							
test appliance (max. 6 weeks)	42	72.4%	0	0%	0	0%	
lateral propulsion wings Somnodent Flex Somnodent Fusion	9 9 0	15.5% 15.5% 0%	28 21 7	48.3% 36.2% 12.1%	12 11 1	57.1% 57.1% 4.8%	
lateral propulsion thrust IST+ IST Classic Narval CC Hamburger UPS	7 6 1 0 0	12.1% 10.3% 1.7% 0% 0%	30 12 1 11 6	51.7% 20.7% 1.7% 19% 10.3%	9 8 0 0 1	42.9% 38.1% 0% 0% 4.8%	
**effective propulsion (mm)**	58	5.92 ( 1.32)	44	6.44 ( 1.48)	19	6.59 ( 1.58)	**0.000**

Abbreviations: T0, pretreatment; T1, after one year; t2, after > 2.5 years; n, number of patients; SD, standard deviation; PSI, periodontal screening index; PSG, polysomnography; PG, polygraphy; AHI, apnea-hypopnea index; no., number; minSaO_2_, minimum oxygen saturation; max., maximum

ANOVA/Friedman was used to compare variables as a function of interval/nominal scaling

*P* <.05 indicates statistically significant differences. Significant *p* values (*p* <.05) are indicated in bold

Clinically, Body Mass Index (BMI), Decayed, Missing, and Filled Teeth (DMFT), number of missing teeth, and PSI showed minimal change over time, while AHI and min SaO_2_ improved significantly (*p* ≤.012).

At the start of MAD therapy, 42 test splints were used and replaced within 6 weeks by 22 splints with lateral thrusts (IST+: *n* = 6; Narval CC: *n* = 11; Hamburger UPS: *n* = 6) and 19 with lateral wings (Somnodent flex: *n* = 12; Somnodent fusion: *n* = 7). The remaining 16 patients received definitive MADs with lateral thrusts (IST+: *n* = 6, IST Classic: *n* = 1) or wings (Somnodent Flex: *n* = 9) without a previous test splint.

At T1 (*n* = 58), 30 patients used lateral thrust devices (IST+: *n* = 12; IST Classic: *n* = 1; Narval CC: *n* = 11; Hamburger UPS: *n* = 6), while 28 had lateral wing devices (Somnodent flex: *n* = 21; Somnodent fusion: *n* = 7). At T2 (*n* = 21), 8 patients used thrust devices (IST+: *n* = 8; Hamburger UPS: *n* = 1) and 12 used wing devices (Somnodent flex: *n* = 11; Somnodent fusion: *n* = 1).

Total MAD protrusion increased significantly over time (F (10.827), *p* =.000), with significant increases from T0 to T1 (*p* =.036) and from T0 to T2 (*p* =.007). Table 1 provides a summary of demographic data.

### Changes in tooth position and migration

In OP2, crown axis changes (inclination, angulation, rotation) and physical tooth translations (mesial/distal, buccal/palatal, intrusion/extrusion) increased overall compared to OP1.

In the maxillary anterior and posterior segments, crowns tended to tilt oro-distally with slight mesial rotation and disto-palatal translation, plus minor extrusion. Conversely, mandibular segments showed a bucco-mesial tilt with slight distal rotation, migration mesio-buccally, and minor extrusion.

Notably, tooth movement was primarily tilting rather than substantial physical translation. Table 2 provides detailed data and the effects of lateral anchorage types (thrust vs. wing designs).

**Table 2:** Changes in 3D tooth movement in the anterior and posterior segments

segment observation period (OP)	n	crown axis [°]			tooth translation [mm]		
		inclination buccal (+) palatal (-)	angulation mesial (+) distal (-)	rotation mesial (+) distal (-)	mesial (+) / distal (-)	buccal (+) palatal (-)	intrusion (+) extrusion (-)
		Mean (SD)	Mean (SD)	Mean (SD)	Mean (SD)	Mean (SD)	Mean (SD)
maxilla all designs							
anterior segment OP1	58	-0.45 (0.94)	-0.32 (0.89)	0.15 (0.78)	-0.07 (0.11)	-0.13 (0.16)	0.01 (0.27)
anterior segment OP2	21	-1.74 (1.38)	-0.66 (0.84)	0.37 (0.91)	-0.20 (0.18)	-0.42 (0.27)	-0.06 (0.40)
posterior segments OP1	58	-0.08 (0.54)	-0.62 (0.98)	0.07 (0.67)	-0.12 (0.21)	0.01 (0.10)	-0.04 (0.36)
posterior segments OP2	21	-0.57 (0.86)	-1.66 (1.22)	0.18 (0.80)	-0.42 (0.30)	-0.04 (0.16)	-0.12 (0.31)
mandible all designs							
anterior segment OP1	58	0.87 (1.55)	0.79 (1.40)	0.20 (1.38)	0.03 (0.31)	0.08 (0.60)	-0.19 (0.48)
anterior segment OP2	21	3.07 (2.47)	1.51 (1.38)	-0.72 (3.32)	0.29 (0.46)	0.67 (1.22)	-0.87 (0.74)
posterior segments OP1	58	0.85 (1.20)	0.63 (1.22)	-0.07 (0.94)	0.04 (0.59)	0.04 (0.28)	-0.12 (0.41)
posterior segments OP2	21	2.43 (1.84)	2.18 (2.41)	-0.22 (1.32)	0.57 (0.95)	0.21 (0.42)	-0.29 (0.62)
maxilla lateral propulsion wings							
anterior segment OP1	28	-0.4 (1.04)	-0.09 (0.81)	-0.00 (0.64)	-0.04 (0.12)	-0.11 (0.19)	-0.04 (0.29)
anterior segment OP2	12	-1.67 (1.52)	-0.63 (0.55)	0.45 (0.42)	-0.21 (0.16)	-0.44 (0.31)	-0.04 (0.29)
posterior segments OP1	28	-0.08 (0.36)	-0.46 (0.88)	0.14 (0.66)	-0.08 (0.18)	-0.02 (0.10)	-0.08 (0.33)
posterior segments OP2	12	-0.44 (0.37)	-1.38 (0.99)	-0.01 (0.58)	-0.33 (0.23)	-0.07 (0.15)	-0.08 (0.33)
maxilla lateral propulsion thrust							
anterior segment OP1	30	-0.45 (0.85)	-0.48 (0.82)	0.24 (0.73)	-0.10 (0.10)	-0.15 (0.13)	0.02 (0.22)
anterior segment OP2	9	-1.73 (1.17)	-0.68 (1.17)	0.22 (1.32)	-0.19 (0.20)	-0.37 (0.22)	0.02 (0.22)
posterior segments OP1	30	-0.01 (0.48)	-0.56 (0.75)	-0.07 (0.54)	-0.13 (0.15)	0.02 (0.06)	-0.04 (0.23)
posterior segments OP2	9	-0.35 (1.04)	-1.12 (1.36)	0.32 (0.71)	-0.31 (0.27)	0.03 (0.09)	-0.04 (0.23)
mandible lateral propulsion wings							
anterior segment OP1	28	0.88 (1.58)	0.78 (1.29)	0.37 (1.69)	0.08 (0.39)	0.16 (0.75)	-0.05 (0.54)
anterior segment OP2	12	3.85 (2.67)	1.51 (1.73)	0.23 (1.44)	0.46 (0.43)	1.24 (0.89)	-0.05 (0.53)
posterior segments OP1	28	0.83 (1.18)	0.55 (0.82)	-0.01 (0.78)	0.15 (0.57)	0.02 (0.26)	-0.08 (0.399
posterior segments OP2	12	2.43 (1.53)	2.20 (2.10)	-0.04 (0.40)	0.92 (0.80)	0.31 (0.31)	-0.08 (0.39)
mandible lateral propulsion thrust							
anterior segment OP1	30	0.91 (1.56)	0.76 (1.52)	0.03 (1.02)	-0.02 (0.21)	-0.02 (0.39)	-0.29 (0.38)
anterior segment OP2	9	1.97 (1.69)	1.48 (0.78)	-1.98 (4.64)	0.05 (0.37)	-0.16 (1.07)	-0.29 (0.38)
posterior segments OP1	30	0.54 (0.69)	0.37 (1.15)	-0.07 (0.62)	-0.06 (0.31)	0.02 (0.14)	-0.19 (0.39)
posterior segments OP2	9	1.38 (1.33)	0.99 (1.51)	-0.41 (1.48)	0.05 (0.37)	-0.06 (0.27)	-0.19 (0.39)

Abbreviations: n, number of patients; SD, standard deviation;

### Comparison of OP1 and OP2

Comparing OP1 and OP2 (Table 3), the maxillary teeth showed a significant increase (*p* ≤.037) in palatal crown inclination, posterior angulation, distal translation, and palatal movement in the anterior segment.

**Table 3:** Statistical comparison of the observation period’s influence on 3-D tooth movements

segment overbiteservation period (OP)	n	crown axis [°]			tooth translation [mm]		
		inclination buccal (+) palatal (-)	angulation mesial (+) distal (-)	rotation mesial (+) distal (-)	mesial (+) / distal (-)	buccal (+) palatal (-)	intrusion (+) extrusion (-)
all designs							
maxilla anterior segment OP1 vs. OP2	21	**0.009**	0.404*	0.237*	**0.037**	**0.002**	0.842
maxilla posterior segments OP1 vs. OP2	21	0.010*	**< 0.001**	0.073*	**0.004**	0.907	0.305*
mandible anterior segment OP1 vs. OP2	21	**< 0.001**	0.068*	0.192*	0.054	0.088	**0.002**
mandible posterior segments OP1 vs. OP2	21	0.004*	**0.004**	0.590*	0.079*	0.243	0.303
lateral propulsion wings							
maxilla anterior segment OP1 vs. OP2	12	0.136	0.084	0.045	0.071	0.060	0.875
maxilla posterior segments OP1 vs. OP2	12	0.136	**0.008**	0.637	0.099	0.784	0.401
mandible anterior segment OP1 vs. OP2	12	**0.002**	0.158	**0.045**	**0.034**	.**010**	**0.008**
mandible posterior segment OP1 vs. OP2	12	0.025	**0.015**	0.182	**0.041**	0.050	0.207
lateral propulsion thrust							
maxilla anterior segment OP1 vs. OP2	9	0.038	0.678	0.953	0.678	0.051	0.889
maxilla posterior segments OP1 vs. OP2	9	0.110	0.214	0.110	0.066	0.594	0.249
mandible anterior segment OP1 vs. OP2	9	0.097	0.214	0.314	0.767	0.594	0.314
mandible posterior segment OP1 vs. OP2	9	0.110	0.173	0.594	0.515	0.173	0.600

Abbreviations: n, number of patients; vs., versus

To analyze the data, the t-test / * Wilcoxon test was used to compare the observation periods depending on the test for normal distribution

*P* <.05 indicates statistically significant differences. Significant *p* values (*p* <.05) are indicated in bold

In the mandible, OP2 had significantly more buccal inclination extrusion in the anterior segment (*p* ≤.004) and mesial tilting in the posterior segment.

Additionally, wing appliances caused greater extrusion (*p* =.003) in the posterior maxilla and more mesio-buccal translation in mandibular segments during OP2 (*p* ≤.023) (Table 4).

**Table 4:** Statistical comparison of the influence of the appliance design on 3D tooth movement

segment observation period (OP)	n	crown axis [°]			tooth translation [mm]		
		inclination buccal (+) palatal (-)	angulation mesial (+) distal (-)	rotation mesial (+) distal (-)	mesial (+) / distal (-)	buccal (+) palatal (-)	intrusion (+) extrusion (-)
		P^‡^	P^‡^	P^‡^	P^‡^	P^‡^	P^‡^
maxilla wing vs. thrust							
maxilla anterior segment OP1	58	0.834	0.162	0.539	0.063	0.497	0.456
maxilla posterior segment OP1	58	0.312	0.807	0.296	0.129	0.167	0.908
maxilla anterior segment OP2	21	0.754	1	0.554	0.702	0.277	0.305
maxilla posterior segment OP2	21	0.095	0.554	0.129	0.917	0.129	**0.003**
mandible wing vs. thrust							
mandible anterior segment OP1	58	0.749	0.559	0.396	0.144	0.661	0.068
mandible posterior segment OP1	58	0.625	0.423	0.631	0.115	0.109	0.446
mandible anterior segment OP2	21	0.111	0.917	0.169	**0.023**	**0.002**	0.331
mandible posterior segment OP2	21	0.082	0.219	0.862	**0.023**	**0.001**	0.228

Abbreviations: n, number of patients; vs., versus

^‡^Differences between wing and thrust design, Mann-Whitney U test used

*P* <.05 indicates statistically significant differences. Significant *p* values (*p* <.05) are indicated in bold

### Changes in overjet and overbite

Overjet and overbite showed a significant decrease over time (Table 5). Overjet decreased significantly (F = 12.16, *p* =.000, ω² = 0.38), notably between T0 and T2 (*p* =.013). Overbite also reduced significantly (F = 10.97, *p* =.000, ω² = 0.35), with differences between T0 and T2 (*p* =.022) and T1 and T2 (*p* =.04).

**Table 5:** Influence of time of investigation and appliance design on overjet and overbite

variable	all designs			lateral propulsion wings			lateral propulsion thrust		
		overjet [mm]	overbite [mm]		overjet [mm]	overbite [mm]		overjet [mm]	overbite [mm]
	n	MW (SD)	MW (SD)	n	MW (SD)	MW (SD)	n	MW (SD)	MW (SD)
time point									
T0	58	2.20 (1.52)	2.53 (1.85)	28	1.82 (1.39)	1.80 (1.52)	30	2.56 (1.55)	3.21 (1.90)
T1	58	1.90 (1.69)	2.42 (1.99)	28	1.57 (1.79)	1.75 (1.89)	30	2.21 (1.55)	3.06 (1.89)
T2	21	0.96 (2.03)	1.19 (1.98)	12	-0.21 (1.44)	0.39 (1.85)	9	2.27 (2.03)	2.26 (1.68)
ANOVA	F P	12.16 **0.000**	10.97 **0.000**		6.024 **0.004**	3.108 0.051		0.355 0.702	0.910 0.407
observation period									
OP 1	58	0.30 (1.05)	0.11 (0.83)	28	0.25 (1.20)	0.55 (0.99)	30	0.34 (0.91)	0.15 (0.67)
OP 2	21	1.21 (1.41)	0.88 (1.11)	12	1.88 (1.29)	1.22 (1.28)	9	0.31 (1.03)	0.42 (0.66)
Wilcoxon	P	**0.006**	**0.002**		**0.005**	**0.006**		0.678	0.086

Abbreviations: n, number of patients; SD, standard deviation

Differences between T0 and T1; the Wilcoxon signed rank test was used to analyze the data

Differences between time points (T0, T1, and T2); ANOVA followed by post hoc Bonferroni was used to analyze the data

*P* <.05 indicates statistically significant differences. Significant *p* values (*p* <.05) are indicated in bold

Wing attachments showed a significant overjet reduction (F = 6.024, *p* =.004) between T0 and T2 (*p* =.004) and T1 and T2 (*p* =.014). All MAD designs significantly reduced both overjet (*p* =.006) and overbite (*p* =.002) between OP1 and OP2, especially with lateral wings (overjet: *p* =.005; overbite: *p* =.006). A significant difference in overjet was found between wing and thrust designs in OP2 (*p* =.009).

### Multiple linear regression analysis

Regression analysis assessed factors influencing overjet and overbite across OP1 and OP2 (b_*0*_): missing teeth (b_*1*_- maxilla/ b_*2*_- mandible), initial PSI (b_*3*_), effective propulsion (b_*4*_), appliance design (b_5_), initial overjet/overbite (b_*6*_).

The full regression equations were:

Overbite/Overjet OP1/OP2 = b_*0*_ + b_*1*_ + b_*2*_ + b_*3*_ + b_*4*_ + b_*5*_ + b_*6*_.

Overbite OP1= -1.95 + (0.02 x Maxillary Missing Teeth) + (-0.05 x Mandibular Missing Teeth) + (0.06 x Initial PSI) + (0.05 x Effective Propulsion) + (-0.19 x Appliance Design) + (0.05 x Initial Overjet / Overbite).

Overjet OP1 = 0.35 + (-0.07 x Maxillary Missing Teeth) + (-0.12 x Mandibular Missing Teeth) + (0.17 x Initial PSI) + (-0.00 x Effective Propulsion) + (-0.15 x Appliance Design) + (0.11 x Initial Overjet / Overbite).

Overbite OP2 = 0.91 + (-0.39 x Maxillary Missing Teeth) + (0.28 x Mandibular Missing Teeth) + (0.43 x Initial PSI) + (-0.11 x Effective Propulsion) + (-1.24 x Appliance Design) + (0.24 x Initial Overjet / Overbite).

Overjet OP2 = 0.74 + (-0.32 x Maxillary Missing Teeth) + (0.25 x Mandibular Missing Teeth) + (0.45 x Initial PSI) + (-2.18 x Effective Propulsion) + (-0.03 x Appliance Design) + (0.28 x Initial Overjet / Overbite).

In OP1, no predictors for overjet/overbite changes were found. In OP2, the model explained 57.14% of the overjet variance (F = 2.67, *p* =.048, R² = 0.57), with appliance design being the main factor (*p* =.005). It explained 43.93% of overbite variance (F = 1.57, *p* =.211, R² = 0.44), also highlighting appliance design as significant (*p* =.047) (Table 6).

**Table 6:** Multiple linear regression analysis of overbite and overjet

dependent variable		Unstandardized coefficients		Standardized coefficients			ANOVA		Adjusted *R*^2^
		Beta	SE	Beta	t	*P*	F	*P*	
observation period 1									
overbite	**Constant**	-0.195	0.80		-0.24	0.810	0.17	0.983	-0.13
	Initially maxillary missing teeth	0.02	0.09	0.04	0.21	0.835			
	Initially mandibular missing teeth	-0.05	0.11	-0.09	-0.42	0.677			
	Initial PSI	0.06	0.17	0.07	0.07	0.706			
	effective propulsion	0.05	0.09	0.08	0.08	0.633			
	appliance design wings/thrust	-0.19	0.32	-0.12	-0.12	0.547			
	initial overjet/overbite	0.05	0.09	0.11	0.11	0.578			
overjet	**Constant**	0.35	0.98		0.35	0.726	0.86	0.582	-0.02
	Initially maxillary missing teeth	-0.07	0.12	-0.11	-0.57	0.572			
	Initially mandibular missing teeth	-0.12	0.13	-0.19	-0.94	0.351			
	Initial PSI	0.17	0.20	0.15	0.85	0.403			
	effective propulsion	-0.00	0.12	-0.00	-0.02	0.982			
	appliance design wings/thrust	-0.15	0.36	-0.07	-0.07	0.684			
	initial overjet/overbite	0.11	0.12	0.16	0.97	0.339			
observation period 2									
overbite	**Constant**	0.91	1.39		0.65	0.528	1.57	0.239	0.16
	Initially maxillary missing teeth	-0.39	0.24	-0.53	-1.65	0.528			
	Initially mandibular missing teeth	0.27	0.26	0.39	1.08	0.125			
	Initial PSI	0.43	0.38	0.29	1.13	0.281			
	effective propulsion	-0.11	0.17	-0.14	-0.62	0.547			
	**appliance design wings/thrust**	-1.24	0.56	-0.53	-2.22	**0.048**			
	initial overjet/overbite	0.24	0.19	0.39	1.29	0.222			
overjet	**Constant**	0.73	1.44		0.51	0.618	**2.67**	**0.048**	0.36
	Initially maxillary missing teeth	-0.32	0.24	-0.35	-1.3	0.217			
	Initially mandibular missing teeth	0.25	0.24	0.29	1.05	0.315			
	Initial PSI	0.45	0.37	0.26	1.22	0.245			
	effective propulsion	-0.03	0,18	-0.04	-0.18	0.863			
	**appliance design wings/thrust**	-2.18	0.63	-0.77	-3.46	**0.005**			
	initial overjet/overbite	0.28	0.25	0.25	1.13	0.28			

Abbreviations: SE, standard error

Significant *p* values (*p* <.05) are indicated in bold

## Discussion

Skeletal changes in long-term MAD wearers are generally secondary to dental shifts [12, 20], and are partly induced by mandibular condyle repositioning in the glenoid fossa [35] and neuromuscular adaptation, similar to orthodontic appliances. Changes in tooth position can generally be induced by low forces of 0.9 to 2.5 N [11], even when applied for only a few hours, e.g. at night [36]. 

Mesial drift of posterior teeth, caused by factors like abrasion [37], ligament contraction [38], and soft tissue pressure is part of the natural tooth migration process. However, these movements occur over a much longer period than MAD-induced changes. For example, sagittal movement of anterior and canine teeth is observed in 25% of people over 6 to 12 years [39], and in over 30% of cases, extrusion of more than 1 mm of natural teeth adjacent to implants occurs over 14 to 20 years [21]. 

The most common changes from long-term MAD use include reduced overjet and overbite [12–15], observed in approximately 86.7% of patients, with posterior open bites in around 50% of users [4, 12, 14, 27]. The reported decrease varies considerably for overjet, with a mean of -0.35 mm [95% CI -0.53, -0.18] for a treatment duration of 1 to 3 years and a mean of -0.77 mm [95% CI -1, -0.51] for a treatment duration of 3 to 5 years, and for overbite, with a mean of -0.31 mm [95% CI -0.48, -0.13] for a treatment duration of 1 to 3 years and a mean of -0.67 mm [95% CI -0.90, -0.44] for a treatment duration of 3 to 5 years [15]. In this study, mean reductions in OP1 were mean 0.30 mm [95% CI] for overjet and mean 0.11 mm [95% CI] for overbite, while in OP2, reductions where 1.21 mm [95% CI ] for overjet and 0.88 mm [95% CI ] for overbite, respectively. These findings indicate a significant decrease in overjet and overbite with treatment duration and consistent with the literature. Wing design resulted in a greater reduction in overjet during OP2 compared to the thrust design.

Individual tooth movements varied widely. Changes over time were inconsistent among individuals. In the wing group, patients with initially negative overjet (*n* = 3), overjet continued to decrease, while overbite decreased in two and increased in one. For patients with initially negative overbite (*n* = 2), both overjet and overbite exhibited variable changes. In the thrust group, initially negative overbite (*n* = 2), both overjet and overbite continued to decrease. Results should be interpreted cautiously due to the small sample size.

MAD design, particularly anterior coverage, can affect overjet and overbite. For example, Ringquist et al. [40] found that a MAD without anterior coverage and 50% maximum mandibular advancement caused only minor reductions of 0.4 mm (from − 1 mm to 0 mm) in overjet and 0.5 mm (from − 1 mm to 0 mm) in overbite over a four years. Conversely, Norrhem et al. [41] reported increased irregularity in lower anterior teeth with flexible appliances lacking incisor coverage. However, research on the impact of anterior coverage is limited. In our study, most splints had full arch coverage, with the Hamburg UPS (which leaves incisors exposed) used infrequently (OP1 *n* = 6, OP2 *n* = 1), likely having minimal impact on the results.

Research on unwanted 3D tooth movement in MAD use is scarce, especially concerning posterior teeth. In this study, unwanted tooth movement was low during OP1 but increased in OP2. Minimal tooth translation in bucco-palatal and mesio-distal directions was noted, primarily due to crown tilting rather than significant shifts. The null hypothesis was thus confirmed. Changes in crown axis and corresponding tooth translations in the anterior segment are reflected in alterations in overjet and overbite. Minor changes in tooth rotation across both segments is expected by the absence of torque with MAD’s full-arch occlusal coverage.

Posterior segment movements included slight extrusion in the maxilla, coupled with a slight mesial and extrusive movement in the mandible. The distal shift of the upper posterior segment aligns with recent literature [10, 12], as is the mesial shift of the lower posterior segment observed in approximately 27% of participants [14, 16–18]. These movements are expected due to the mechanical forces acting on the entire dental arch [17]. 

Studies have reported reduced posterior occlusal contacts [14, 25, 42] with posterior open bites in up to 11.94% of patients after approximately 7 months [27, 43]. Consistent with our findings, posterior tooth extrusion [17] occurred only after prolonged MAD wear (over 24 months) [22]. The observation of a posterior open bite with molar extrusion is not inherently contradictory. Molar extrusion can result from a lack of occlusal support due to a posterior open bite caused by premature anterior contacts after overjet reduction, leading to mandibular rotation [44]. 

Studies have shown a greater increase in mandibular arch width (0.2 to 0.66 mm) compared to maxillary arch width (0.03 to 0.6 mm) [16]. Accordingly, in this study, mandibular arch width increased of approximately 0.21 ± 0.42 mm at T2, whereas maxillary arch width tended to decrease by 0.04 ± 0.16 mm at T2.

Finite element analyses (FEA) show that the connecting mechanism of upper and lower appliances affects the loads on the periodontal ligaments and the teeth [12]. Appliances employing anterior propulsion (e.g., an anterior reverse connecting rod) create higher stresses (4.26 kPa) compared to those with bilateral propulsion (thrust, wings), which generate lower stresses (3.56 kPa) [12]. Appliances with lateral propulsion tend to distribute stresses more evenly, with a concentration at the molars [12, 45]. Wing appliances showed slightly lower maximum stresses than thrust appliances, recording 3.27 kPa at the periodontal ligament and 287 kPa at the teeth, compared to 3.56 kPa at the periodontal ligament and 302 kPa at the teeth for thrust appliances [12]. In both designs, stresses were concentrated in the coronal portion of the periodontal ligament, affecting the medio-distal cervical region. Thrust appliances impacted the maxillary molars and mandibular premolars, while wing appliances showed stresses on the maxillary molars and mandibular second premolars, depending on the splint’s wing position [12]. 

This study found a significantly greater reduction in overjet and overbite, and more extrusion of maxillary teeth with wing appliances, along with mostly buccal tooth translation in the posterior mandibular segment. Despite significant differences in overjet and overbite reduction, the differences in posterior maxilla translation were under 0.1 mm, raising questions about clinical relevance. In the mandible, results were similar to FEA findings [12], though caution is needed due to less precise measurement methods. Patient adherence to MAD usage was not consistently assessed, which could affect efficacy and dental side effects [27, 46]. 

MAD material may also impact overjet and overbite, with softer materials with less protrusion (< 6 mm) yielding milder changes than hard acrylic materials over an observation period of approximately 2.5 ± 0.5 years [28]. In the present study, 42 patients were fitted with a test splint made of soft elastomer for the first 6 weeks, which may have influenced overjet and overbite. The wing appliance used (Somnodent) features a soft inner liner (b-flex Comfort Liner), which may promote more tooth movement than the MADs in the Marklund et al.’s study [28]. The greater tooth movement observed with the wing appliance compared to thrust appliances in Bruno et al.‘s [12] FEA study may be due to the fact that their splint model of the Somnodent splint did not have a soft liner and was made of homogeneous acrylic. Despite this, literature on soft splints is limited. In temporomandibular dysfunction therapy, hard splints are generally referred for easier adjustment, better hygiene, reduced risk of unintended tooth movement, and enhanced durability [47]. 

The extent of undesirable changes in the dentition caused by MAD therapy is closely tied to the degree of protrusion and moderately to treatment duration [13]. Higher protrusion exerts greater forces on the periodontal ligament, particularly when exceeding 50% protrusion [5, 12]. Cohen-Levy et al. demonstrated a near-linear relationship between force and adverse events, with forces reaching about 1.18 N per mm of advancement. In this study, protrusion exceeded 50%, with a mean of 5.92 ± 1.32 mm at T0, 6.44 ± 1.48 mm at T1, and 6.59 ± 1.58 mm at T2, likely contributing to tooth movement, although no direct link with changes in overjet and overbite was found. Thus the null hypothesis was rejected. For protrusions over 70%, FE analyses showed periodontal ligament pressures above 4.7 kPa, linked to root resorption risk [12]. As tooth movement depends on MAD-applied forces on the periodontal ligament, the dentition’s resistance significantly influences tooth position changes. Therefore, partial tooth loss and severe periodontal disease are relative contraindications for MAD therapy, as they may impair force distribution [12]. 

From a clinical perspective, the measured changes in tooth position over the study period are relatively small, yet they do influence bite position. Given the occlusal tactility threshold of natural antagonists (16 ± 9 μm [48]), patients may not consciously perceive these changes, as gradual adaptations often go unnoticed or are well tolerated [18, 26]. However, the progressive tilting of the dental arches can affect the overall mandibular advancement and efficacy of the appliance. This highlights the need for regular follow-up assessments, including PG/PSG evaluations, to monitor changes and adjust titration as needed. Furthermore, the study underscores the value of digital superimposition for precise 3D monitoring of tooth movement, aiding dentists in optimizing MAD therapy while minimizing unwanted occlusal alterations.

### Limitations

This study has limitations. Only 21 data sets were available at T2, leading to a small sample size in OP2, and the subdivision by MAD design complicates interpretation. In OP1, 42 out of 58 patients wore a temporary test splint for up to 6 weeks, which likely had minimal impact on outcomes; however, the analysis did not differentiate between temporary and permanent splints, possibly influencing results. Patients heterogeneity also limits predictive value of the findings. Methodologically, while palatal vault surface registration is standard for maxilla superimposition, inconsistent mandibular anatomy may reduce measurement accuracy. The absence of a control group also limits the assessment of physiological tooth movement. The digital superimposition technique in this study provides a more accurate 3D assessment of tooth position changes than conventional methods but does not capture other possible MAD therapy side effects, such as temporomandibular joint issues, intraoral tissue reactions, tooth or restoration damage, and appliance-related problems. Additionally, the effect of using morning occlusal guides on addressing mesial displacement of mandibular canines and molars [49] could not be assessed due to insufficient documentation.

## Conclusions

The results of this study led to the following conclusions:

1. Long-term MAD use significantly affects 3D tooth movement, leading to mesio-occlusion, anterior open bite, and decreased occlusal contacts.
2. Unwanted tooth movements appear to be tilting of the entire row rather than translation or bodily movement.
3. Wing appliances appear to cause more changes in tooth position than thrust appliances; however, clinical differences are negligible.
4. Digital superimposition provides a precise 3D assessment of tooth position changes, particularly in the maxilla, aiding orthodontic evaluation.

## Data Availability

All data generated or analyzed during this study are included in this published article and can be made available on request.

## Abbreviations

3D: Three dimensional
AHI: Apnea hypopnea index
BMI: Body mass index
CPAP: Continuous positive airway pressure
DMFT: Decayed, missing, and filled teeth
FEA: Finite element analyses
MAD: Mandibular advancement device
OP1,2: Observation period 1, 2
OSA: Obstructive sleep apnea
PSG: Polysomnography
PG: Polygraphy
SaO_2_: Oxygen saturation
STL: Standard tessellation language
